# Poly[tetra­aquadi-μ_4_-oxalato-potassium­ytterbium(III)]

**DOI:** 10.1107/S1600536811046022

**Published:** 2011-11-09

**Authors:** Feng-Ming Zhang, Guang-Feng Hou, Peng-Fei Yan, Guang-Ming Li

**Affiliations:** a Key Laboratory of Functional Inorganic Materials Chemistry (MOE), School of Chemistry and Materials Science, Heilongjiang University, Harbin 150080, People’s Republic of China

## Abstract

In the title compound, [KYb(C_2_O_4_)_2_(H_2_O)_4_]_*n*_, the Yb^III^ ion lies on a site of 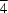 symmetry in a dodeca­hedral environment defined by eight O atoms from four oxalate ligands. The K atom lies on a different 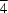 axis and is coordinated by four O atoms from four oxalate ligands and four water O atoms. The oxalate ligand has an inversion center at the mid-point of the C—C bond. The metal ions are linked by the oxalate ligands into a three-dimensional framework. O—H⋯O hydrogen bonding is present in the crystal structure.

## Related literature

For related structures, see: Camara *et al.* (2003 ▶); Zhang *et al.* (2009 ▶).
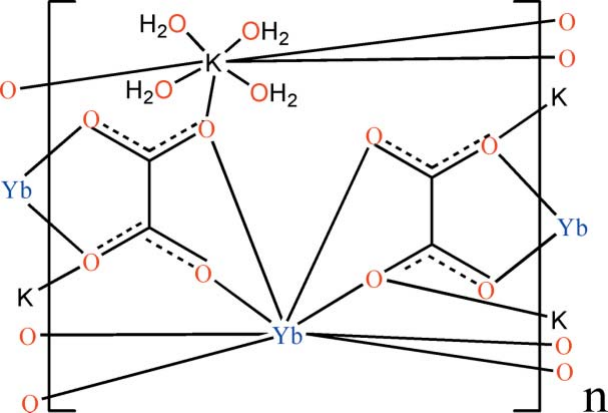


## Experimental

### 

#### Crystal data

[KYb(C_2_O_4_)_2_(H_2_O)_4_]
*M* *_r_* = 460.24
Tetragonal, 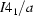

*a* = 11.3502 (16) Å
*c* = 8.9142 (18) Å
*V* = 1148.4 (3) Å^3^
*Z* = 4
Mo *K*α radiation
μ = 8.57 mm^−1^
*T* = 293 K
0.08 × 0.07 × 0.07 mm

#### Data collection

Rigaku R-AXIS RAPID diffractometer
Absorption correction: multi-scan (*ABSCOR*; Higashi, 1995 ▶) *T*_min_ = 0.562, *T*_max_ = 0.606
5407 measured reflections
648 independent reflections
585 reflections with *I* > 2σ(*I*)
*R*_int_ = 0.051

#### Refinement

*R*[*F* ^2^ > 2σ(*F* ^2^)] = 0.017
*wR*(*F* ^2^) = 0.039
*S* = 0.94
648 reflections
41 parameters
3 restraints
H-atom parameters constrained
Δρ_max_ = 0.55 e Å^−3^
Δρ_min_ = −0.43 e Å^−3^

### 

Data collection: *RAPID-AUTO* (Rigaku, 1998 ▶); cell refinement: *RAPID-AUTO*; data reduction: *CrystalStructure* (Rigaku/MSC, 2002 ▶); program(s) used to solve structure: *SHELXS97* (Sheldrick, 2008 ▶); program(s) used to refine structure: *SHELXL97* (Sheldrick, 2008 ▶); molecular graphics: *DIAMOND* (Brandenburg, 1999 ▶); software used to prepare material for publication: *SHELXL97*.

## Supplementary Material

Crystal structure: contains datablock(s) I, global. DOI: 10.1107/S1600536811046022/hy2482sup1.cif

Structure factors: contains datablock(s) I. DOI: 10.1107/S1600536811046022/hy2482Isup2.hkl

Additional supplementary materials:  crystallographic information; 3D view; checkCIF report

## Figures and Tables

**Table 1:** Selected bond lengths (Å)

K1—O1	2.8402 (19)
K1—O3	2.871 (3)
Yb1—O1	2.3629 (19)
Yb1—O2^i^	2.304 (2)

Symmetry code: (i) 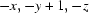.

**Table 2:** Hydrogen-bond geometry (Å, °)

*D*—H⋯*A*	*D*—H	H⋯*A*	*D*⋯*A*	*D*—H⋯*A*
O3—H1⋯O3^ii^	0.85	2.08	2.899 (3)	163
O3—H2⋯O2^iii^	0.85	2.06	2.837 (3)	152

Symmetry codes: (ii) 
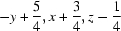
; (iii) 
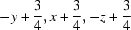
.

**Fig. 1.**
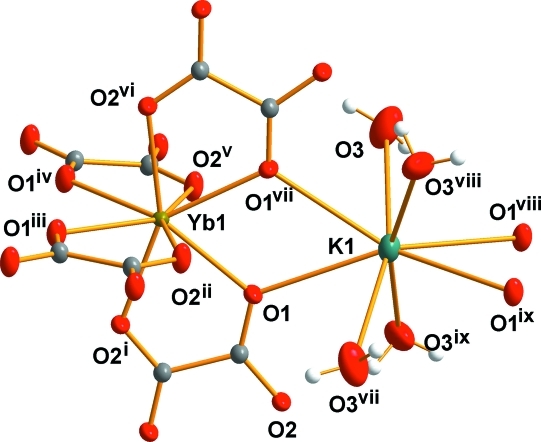
The asymmetric unit of the title compound, showing displacement ellipsoids at the 50% probability level. [Symmetry codes: (i) -x, 1-y, -z; (ii) 1/4-y, 3/4+x, -1/4+z; (iii) -3/4+y, 3/4-x, -1/4-z; (iv) 3/4-y, 3/4+x, -1/4-z; (v) -1/4+y, 3/4-x, -1/4+z; (vi) x, 1/2+y, z; (vii) -x, 3/2-y, z; (viii) 3/4-y, 3/4+x, 3/4-z; (ix) -3/4+y, 3/4-x, 3/4-z.]

**Fig. 2.**
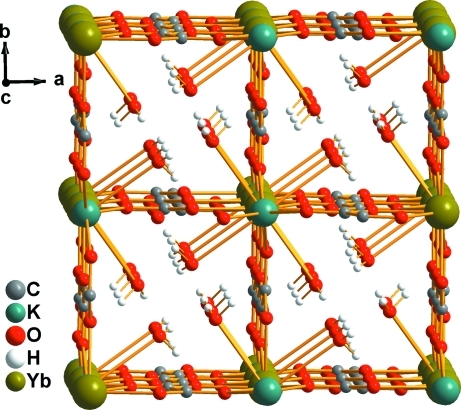
A packing view along [001], showing the three-dimensional framework.

## supplementary crystallographic information

### Comment

Lanthanide complexes with spectroscopic and magnetic properties are currently of considerable interest. Oxalate ligand can serve as bridging ligand in high dimensional frameworks (Camara *et al.*, 2003; Zhang *et al.*, 2009). In this paper, we present the synthesis and crystal structure of the title compound.

The title compound was obtained as a byproduct by the decomposition of 1,3,5-triazine-2,4,6-tricarboxylate ligand. In the title compound, [YbK(C_2_O_4_)_2_(H_2_O)_4_]_n_, the eight-coordinated Yb^III^ ion lies on a 4 site symmetry in a distorted dodecahedral geometry defined by eight O atoms from four oxalate ligands. The eight-coordinated K ion is also locate on another site of 4 symmetry in a distorted dodecahedral geometry defined by four O atoms from oxalate ligands and four O atoms from water molecules (Fig. 1, Table 1).

In the crystal, each oxalate ligand links two Yb and two K atoms, forming a three-dimensional framework (Fig. 2). O—H···O hydrogen bonds are present (Table 2).

### Experimental

The title compound was obtained as a byproduct caused by the decomposition of 1,3,5-triazine-2,4,6-tricarboxylate ligand. Yb(NO_3_)_3._6H_2_O (14.01 mg, 0.03 mmol) and potassium salt of 1,3,5-triazine-2,4,6-tricarboxylate (9.8 mg, 0.03 mmol) were dissolved in 15 ml water. After stirring at room temperature for 0.5 h, the solution was allowed to stand for about one week. Colorless block crystals were obtained in 36% yield.

### Refinement

Water H atoms were initially located in a differece Fourier map and were treated as riding atoms, with O—H = 0.85 Å and with *U*_iso_(H) = 1.5*U*_eq_(O).

### Crystal data

**Table:**

[KYb(C_2_O_4_)_2_(H_2_O)_4_]	*D*_x_ = 2.662 Mg m^−^^3^
*M**_r_* = 460.24	Mo *K*α radiation, λ = 0.71073 Å
Tetragonal, *I*4_1_/*a*	Cell parameters from 4696 reflections
Hall symbol: -I 4ad	θ = 3.6–27.4°
*a* = 11.3502 (16) Å	µ = 8.57 mm^−^^1^
*c* = 8.9142 (18) Å	*T* = 293 K
*V* = 1148.4 (3) Å^3^	Block, colorless
*Z* = 4	0.08 × 0.07 × 0.07 mm
*F*(000) = 868	

### Data collection

**Table:**

Rigaku R-AXIS RAPID diffractometer	648 independent reflections
Radiation source: fine-focus sealed tube	585 reflections with *I* > 2σ(*I*)
graphite	*R*_int_ = 0.051
ω scan	θ_max_ = 27.4°, θ_min_ = 3.6°
Absorption correction: multi-scan (*ABSCOR*; Higashi, 1995)	*h* = −14→14
*T*_min_ = 0.562, *T*_max_ = 0.606	*k* = −13→14
5407 measured reflections	*l* = −10→11

### Refinement

**Table:**

Refinement on *F*^2^	Primary atom site location: structure-invariant direct methods
Least-squares matrix: full	Secondary atom site location: difference Fourier map
*R*[*F*^2^ > 2σ(*F*^2^)] = 0.017	Hydrogen site location: inferred from neighbouring sites
*wR*(*F*^2^) = 0.039	H-atom parameters constrained
*S* = 0.94	*w* = 1/[σ^2^(*F*_o_^2^) + (0.*P*)^2^] where *P* = (*F*_o_^2^ + 2*F*_c_^2^)/3
648 reflections	(Δ/σ)_max_ < 0.001
41 parameters	Δρ_max_ = 0.55 e Å^−^^3^
3 restraints	Δρ_min_ = −0.43 e Å^−^^3^

### Fractional atomic coordinates and isotropic or equivalent isotropic displacement parameters (Å^2^)

**Table:**

	*x*	*y*	*z*	*U*_iso_*/*U*_eq_	
C1	0.0038 (3)	0.5244 (3)	0.0802 (3)	0.0159 (6)	
K1	0.0000	0.7500	0.3750	0.0296 (4)	
O1	0.0084 (2)	0.63272 (19)	0.0937 (2)	0.0209 (5)	
O2	0.0045 (2)	0.44765 (18)	0.1836 (2)	0.0224 (5)	
O3	0.2057 (3)	0.8934 (3)	0.3322 (3)	0.0509 (8)	
H1	0.2363	0.9125	0.2486	0.076*	
H2	0.2520	0.8473	0.3787	0.076*	
Yb1	0.0000	0.7500	−0.1250	0.01065 (10)	

### Atomic displacement parameters (Å^2^)

**Table:**

	*U*^11^	*U*^22^	*U*^33^	*U*^12^	*U*^13^	*U*^23^
C1	0.0188 (16)	0.0154 (15)	0.0136 (12)	0.0004 (12)	−0.0010 (11)	0.0004 (11)
K1	0.0345 (6)	0.0345 (6)	0.0198 (7)	0.000	0.000	0.000
O1	0.0346 (14)	0.0125 (11)	0.0156 (9)	−0.0003 (10)	−0.0013 (8)	−0.0017 (8)
O2	0.0399 (14)	0.0122 (11)	0.0150 (9)	−0.0010 (10)	−0.0009 (9)	−0.0006 (8)
O3	0.0374 (17)	0.075 (2)	0.0402 (13)	0.0106 (16)	−0.0038 (12)	0.0195 (15)
Yb1	0.01004 (12)	0.01004 (12)	0.01186 (15)	0.000	0.000	0.000

### Geometric parameters (Å, °)

**Table:**

C1—O1	1.236 (4)	Yb1—O1	2.3629 (19)
C1—O2	1.269 (4)	Yb1—O2^i^	2.304 (2)
C1—C1^i^	1.536 (5)	O3—H1	0.8500
K1—O1	2.8402 (19)	O3—H2	0.8499
K1—O3	2.871 (3)		
			
O1—C1—O2	127.7 (3)	O2^i^—Yb1—O1^v^	137.29 (7)
O1—C1—C1^i^	116.9 (3)	O2^ii^—Yb1—O1^vi^	137.29 (7)
O2—C1—C1^i^	115.4 (3)	O2^iii^—Yb1—O1^vi^	82.21 (8)
O1—K1—O3	96.95 (7)	O2^iv^—Yb1—O1^vi^	68.87 (7)
C1—O1—Yb1	118.53 (17)	O2^i^—Yb1—O1^vi^	76.19 (8)
C1—O1—K1	123.44 (17)	O1^v^—Yb1—O1^vi^	132.92 (6)
Yb1—O1—K1	117.59 (8)	O2^ii^—Yb1—O1	76.19 (8)
C1—O2—Yb1^i^	120.27 (18)	O2^iii^—Yb1—O1	137.29 (7)
K1—O3—H1	126.4	O2^iv^—Yb1—O1	82.21 (8)
K1—O3—H2	95.1	O2^i^—Yb1—O1	68.87 (7)
H1—O3—H2	109.3	O1^v^—Yb1—O1	68.77 (10)
O2^ii^—Yb1—O2^iii^	92.95 (2)	O1^vi^—Yb1—O1	132.92 (6)
O2^ii^—Yb1—O2^iv^	153.79 (9)	O2^ii^—Yb1—O1^vii^	68.87 (7)
O2^iii^—Yb1—O2^iv^	92.95 (2)	O2^iii^—Yb1—O1^vii^	76.19 (8)
O2^ii^—Yb1—O2^i^	92.95 (2)	O2^iv^—Yb1—O1^vii^	137.29 (7)
O2^iii^—Yb1—O2^i^	153.79 (9)	O2^i^—Yb1—O1^vii^	82.21 (8)
O2^iv^—Yb1—O2^i^	92.95 (2)	O1^v^—Yb1—O1^vii^	132.92 (6)
O2^ii^—Yb1—O1^v^	82.21 (8)	O1^vi^—Yb1—O1^vii^	68.77 (10)
O2^iii^—Yb1—O1^v^	68.87 (7)	O1—Yb1—O1^vii^	132.92 (6)
O2^iv^—Yb1—O1^v^	76.19 (8)		

Symmetry codes: (i) −*x*, −*y*+1, −*z*; (ii) *y*−1/4, −*x*+3/4, *z*−1/4; (iii) *x*, *y*+1/2, −*z*; (iv) −*y*+1/4, *x*+3/4, *z*−1/4; (v) −*x*, −*y*+3/2, *z*; (vi) *y*−3/4, −*x*+3/4, −*z*−1/4; (vii) −*y*+3/4, *x*+3/4, −*z*−1/4.

### Hydrogen-bond geometry (Å, °)

**Table:**

*D*—H···*A*	*D*—H	H···*A*	*D*···*A*	*D*—H···*A*
O3—H1···O3^viii^	0.85	2.08	2.899 (3)	163
O3—H2···O2^ix^	0.85	2.06	2.837 (3)	152

Symmetry codes: (viii) −*y*+5/4, *x*+3/4, *z*−1/4; (ix) −*y*+3/4, *x*+3/4, −*z*+3/4.
